# Epidemiology of Lyme Disease, Nova Scotia, Canada, 2002–2013

**DOI:** 10.3201/eid2110.141640

**Published:** 2015-10

**Authors:** Todd F. Hatchette, B. Lynn Johnston, Emily Schleihauf, Angela Mask, David Haldane, Michael Drebot, Maureen Baikie, Teri J. Cole, Sarah Fleming, Richard Gould, Robbin Lindsay

**Affiliations:** Nova Scotia Health Authority, Halifax, Nova Scotia, Canada (T.F. Hatchette, B.L. Johnston, D. Haldane);; Dalhousie University, Halifax (T.F. Hatchette, B.L. Johnston, D. Haldane); Public Health Agency of Canada, Ottawa, Ontario, Canada (E. Schleihauf, A. Mask);; Public Health Agency of Canada, Winnipeg, Manitoba, Canada (M. Drebot, R. Lindsay) Government of Nunavut, Iqaluit, Nunavut, Canada (M. Baikie);; Nova Scotia Department of Health and Wellness, Halifax (T.J. Cole, S. Fleming);; York Region Public Health, Newmarket, Ontario, Canada (R. Gould)

**Keywords:** Lyme disease, Nova Scotia, Canada, seroprevalence, Borrelia burgdorferi, epidemiology, vector-borne infections, ticks

## Abstract

*Ixodes scapularis* ticks, which transmit *Borrelia* *burgdorferi*, the causative agent of Lyme disease (LD), are endemic to at least 6 regions of Nova Scotia, Canada. To assess the epidemiology and prevalence of LD in Nova Scotia, we analyzed data from 329 persons with LD reported in Nova Scotia during 2002–2013. Most patients reported symptoms of early localized infection with rash (89.7%), influenza-like illness (69.6%), or both; clinician-diagnosed erythema migrans was documented for 53.2%. In a separate serosurvey, of 1,855 serum samples screened for antibodies to *B.* *burgdorferi*, 2 were borderline positive (both with an indeterminate IgG on Western blot), resulting in an estimated seroprevalence of 0.14% (95% CI 0.02%–0.51%). Although LD incidence in Nova Scotia has risen sharply since 2002 and is the highest in Canada (16/100,000 population in 2013), the estimated number of residents with evidence of infection is low, and risk is localized to currently identified LD-endemic regions.

## Abstract 2

Nova Scotia has the highest reported incidence in Canada, but risk is localized to identified disease-endemic regions.

Lyme disease (LD) is an emerging vector-borne infection caused by *Borrelia burgdorferi*, which is transmitted to humans by infected ticks. In Nova Scotia, Canada, the vector is the blacklegged tick, *Ixodes scapularis*. Approximately 300,000 cases of LD occur in the United States each year (*1*). In Canada, infected *Ixodes* ticks are now endemic to parts of British Columbia (*I. pacificus*), Manitoba, Ontario, Quebec, New Brunswick, and Nova Scotia (*I. scapularis*) (*2*). The number of Canadians with LD has risen since LD became nationally reportable in 2009; a total of 682 cases from across the country were reported to the Public Health Agency of Canada (PHAC) in 2013, which most likely underrepresents the true number (*2*–*4*). As the geographic range of *I. scapularis* ticks expands, more Canadians will be at risk for LD.

Since the first case of locally acquired LD was reported in 2002, the number of human infections in Nova Scotia has risen sharply; 154 cases were reported in 2013 alone. LD can manifest with localized disease, most commonly erythema migrans (EM), or disseminated illness with neurologic, cardiac, and/or joint involvement. In the United States, up to 7% of cases are asymptomatic (*5*). In Canada, tick surveillance, coordinated and conducted by the Nova Scotia Departments of Health and Wellness and Natural Resources and the National Microbiology Laboratory (NML) of the PHAC, has identified the establishment of infected blacklegged tick populations in 6 regions in Nova Scotia, and these ticks have been found sporadically in many other locations, suggesting potential LD risk across the province. However, contemporary LD risk has been difficult to quantify in Nova Scotia because of the dynamic and expanding nature of vector tick populations, occurrence of missed and/or asymptomatic infections, and changes in surveillance methods and case definitions. Here we describe the epidemiology of LD and the results of a 2012 provincial serosurvey for antibodies to *B. burgdorferi* to characterize and estimate the risk for features of LD in Nova Scotia.

## Methods

### LD Case Data

LD is reportable in Nova Scotia. Case data were provided by Population Health Assessment and Surveillance, Nova Scotia Department of Health and Wellness (Table 1). Public health nurses investigate all LD cases by follow-up with the health care provider and patient. Data presented were extracted from case report forms. Numbers of tests conducted by the Capital District Health Authority Division of Microbiology, the Nova Scotia testing site for LD, were extracted from the laboratory information system. Regions to which LD is endemic (endemic regions) are defined by using the Canadian national definition. A confirmed endemic region is one in which active field surveillance has identified a reproducing population of ticks confirmed by the presence of all 3 stages on resident animals or in the environment for at least 2 consecutive years, and *B. burgdorferi* is detected in these ticks and/or wild animal hosts in the locality (*2**,**6*). For the last 2 regions identified, all 3 stages were identified in 1 year only.

**Table 1:** LD case definitions used in Nova Scotia, Canada*

Period	Case classification	Case definition
2002–2007	Confirmed	EM or other clinical evidence and laboratory evidence of infection (based on CDC criteria) (*8*)
2008–2015	Confirmed	Clinical evidence of illness with a history of residence in, or visit to, an endemic region and laboratory evidence of infection (*8*)
2008–2015	Probable	Clinical evidence of illness without a history of residence in, or visit to, an endemic region and laboratory evidence of infection (*8*)
		OR clinician-observed EM without laboratory evidence but with history of residence in, or visit to, an endemic region

*CDC, Centers for Disease Control and Prevention; EM, erythema migrans; LD, Lyme disease.

### Serosurvey

We used samples of residual serum from specimens submitted for diagnostic testing that would otherwise have been discarded. Aliquots of residual serum submitted for prenatal screening, electrolyte testing, cholesterol testing, or HIV screening were collected from regional laboratories during May 1–August 30, 2012. Each serum sample was deidentified so that it could not be linked to a specific patient. These specimens were chosen with the intent of obtaining samples from healthy persons undergoing blood tests as part of a regular health check-up. Serum samples were stratified by patient age, sex, and District Health Authority (DHA), and sampling was proportionate to the Nova Scotia population in five 10-year age groups for ages 10–59 years and 1 age group for ages 60–64 years. The Research Ethics Board of each DHA approved the serosurvey. One Research Ethics Board required an opt-out option for patients undergoing blood collection during the study period, achieved by publicizing the study using posters and asking patients who did not want their serum used to identify themselves at the time of collection. No patients opted out.

### Serologic Testing

We detected antibodies to *B. burgdorferi* using a commercially available enzyme immunoassay (EIA) that used a whole-cell sonicate of *B. burgdorferi* (*B. burgdorferi* ELISA II, Wampole Laboratories, Princeton, NJ, USA). Samples were tested at the Capital DHA microbiology laboratory. In accordance with Canadian and US guidelines (*7*,*8*), specimens that screened positive or equivocal on the EIA were sent to the NML for confirmatory testing. The NML uses a 2-tiered approach whereby specimens that test positive or equivocal by EIA are retested by using C6 ELISA (Immunetics, Boston, MA, USA) and Western blot (Euroimmun, Morris Plains, NJ, USA). Consistent with criteria from the US Centers for Disease Control and Prevention, a positive IgG Western blot (WB) (5 of 10 significant bands reactive) was considered conclusive evidence of previous infection (*9*). In addition, the NML has created a borderline category for serum for which 4 of 10 reactive significant bands are documented but includes an additional fifth band that is visible on the blot but is insufficiently intense to be considered reactive.

### Statistics

**Figure 1:**
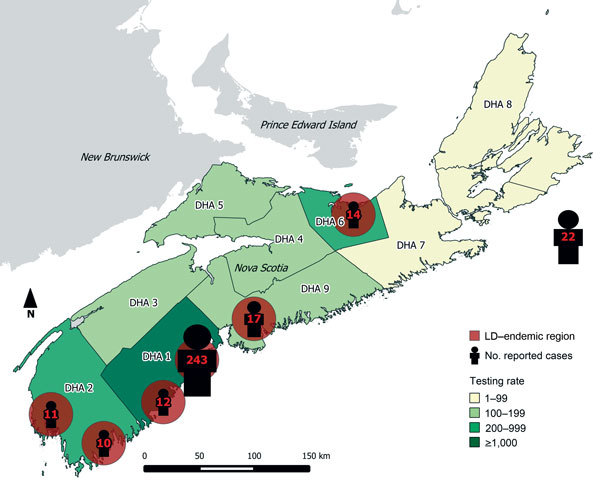
Reported cases of Lyme disease (LD) for 2002–2013, by endemic region of exposure and LD testing rates by District Health Authority (DHA) for 2013, Nova Scotia, Canada. Of the 22 cases without a known link to an LD-endemic area in Nova Scotia, 17 persons were infected outside the province (Europe and the United States); for 5 persons, location of exposure was either unknown or outside of known LD-endemic regions. Testing rate is per 100,000 population.

We calculated sample size for the serosurvey on the basis of an estimated seroprevalence of 1.0% ± 0.5% precision within a 95% CI, with oversampling in DHA 1, DHA 2, and DHA 3, where greater LD activity has been noted (Figure 1). Descriptive statistics were used to characterize reported LD cases and testing results from the serosurvey. Seroprevalence estimates were produced and 95% CIs calculated by using the Clopper-Pearson Exact method. Design weights accounted for regional oversampling in the provincial estimates. Statistical analyses were conducted by using SAS version 9.4 (SAS Institute, Inc., Cary, NC, USA). Maps were created by using QGIS 2.0 (http://qgis.org/en/site/). Population estimates were based on 2011 census data from Statistics Canada (*10*).

## Results

### Endemic Regions

The first established *B. burgdorferi*–infected blacklegged tick population in Nova Scotia was identified in a rural region within DHA 1 in 2003. The second endemic region was identified in a park within the largest urban center in DHA 9 in 2006. In 2008, a rural region within DHA 2 was declared to be endemic, and in 2010, a rural region in DHA 6 was declared endemic. In 2011 and 2012 the fifth and sixth endemic regions were identified in other rural regions within DHAs 1 and 2, >20 km away from regions previously identified.

### LD Cases

**Figure 2:**
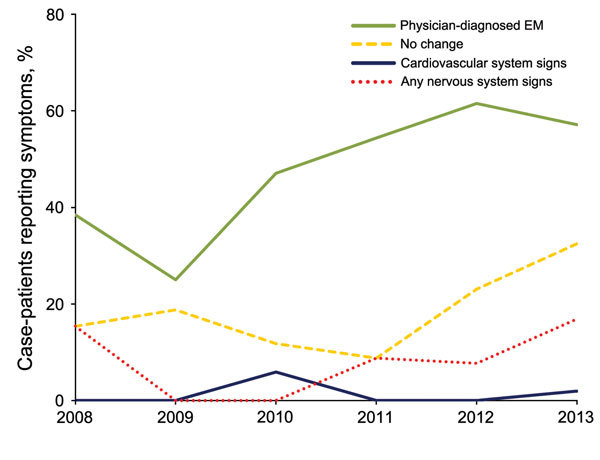
Percentage of Lyme disease (LD) case-patients with symptom complex, by year, Nova Scotia, Canada, 2008–2013. Cardiovascular system signs include atrioventricular block, mycarditis, and other. Nervous system signs comprise peripheral or central signs. EM, erythema migrans.

A total of 329 LD cases were reported in Nova Scotia during 2002–2013. Case-patients were a median of 56 (range 0–85) years of age, and most (76.9%) were male (Table 2). Only 26.4% of LD patients reported a definite history of a tick bite. Most reported symptoms of early localized LD, including influenza-like illness and EM or other non-EM rash. Physician-diagnosed EM was reported for 53.2% of cases. Central clearing (i.e., reduction or of the erythema near the center of the rash) was reported for 49.7%; a total of 33.7% reported no central clearing, and for 16.6%, central clearing was unknown. A total of 125 LD patients had early disseminated or late infection, including recurrent joint swelling and Bell palsy. The percentage of cases with clinician-observed EM increased over time, and brief recurrent joint swelling was reported in a greater percentage of patients in 2013 than previously (Figure 2). Thirteen LD patients were hospitalized (Table 3), but the reasons for admission were unknown.

**Table 2:** Characteristics of 329 reported LD case-patients, Nova Scotia Canada, 2002–2013*

Characteristic	No. (%)
Sex	
M	200 (60.8)
F	129 (39.2)
Age group, y	
0–9	31 (9.4)
10–19	33 (10.0)
20–29	17 (5.2)
30–39	17 (5.2)
40–49	32 (9.7)
50–59	67 (20.4)
60–69	76 (23.1)
70–79	44 (13.4)
>80	12 (3.6)
Case definition met	
Confirmed	241 (73.3)
Probable	
EM with history of exposure to endemic region	66 (20.1)
Clinical evidence of illness without exposure to endemic region with laboratory evidence	22 (6.7)
Symptom†	
Rash, any reported	295 (89.7)
Influenza-like illness	229 (69.6)
EM, physician-diagnosed	175 (53.2)
Recurrent brief episodes of large joint swelling	77 (23.4)
Bell palsy	23 (7.0)
Nervous system symptoms, excluding Bell palsy	21 (6.4)
Cardiovascular system signs	4 (1.2)
History of tick bite	
Definite	87 (26.4)
Possible, exposure to wooded or brushy regions	199 (60.5)
Unknown	43 (13.1)
DHA of residence‡	
DHA 1	235 (71.4)
DHA 2	21 (6.4)
DHA 3	9 (2.7)
DHA 4	4 (1.2)
DHA 5	1 (0.3)
DHA 6	8 (2.4)
DHA 7	4 (1.2)
DHA 8	2 (0.6)
DHA 9	45 (13.7)
Hospitalized	13 (4.0)

*Endemic regions as defined in June 2014. Some cases classified to exposure retrospectively (i.e., case occurred before endemic region were declared). DHA, District Health Authority; EM, erythema migrans; LD, Lyme disease. †More than 1 symptom might be reported per case-patient. Rash, any reported, includes EM. Cardiovascular system signs include atrioventricular block, mycarditis, other. ‡DHA 1, endemic regions declared in 2003 and 2012; DHA 2, endemic regions declared in 2008 and 2011; DHA 6, endemic region declared in 2010; DHA 9, endemic region declared in 2006.

**Table 3:** Characteristics of reported LD in 13 hospitalized patients, Nova Scotia Canada, 2002–2013*

Characteristic	No. (%)
Sex	
M	10 (76.9)
F	3 (23.1)
Age group, y	
0–9	2 (15.4)
10–19	2 (15.4)
20–29	2 (15.4)
30–39	2 (15.4)
40–49	1 (7.7)
50–59	1 (7.7)
60–69	1 (7.7)
70–79	1 (7.7)
>80	1 (7.7)
Symptoms†	
Rash, any reported	4 (30.8)
Influenza-like illness	9 (69.2)
EM, physician-diagnosed	4 (30.8)
Brief recurrent joint swelling	2 (15.4)
Bell palsy	3 (23.1)
Any nervous system sign or symptom	2 (15.4)
Cardiovascular system signs	2 (15.4)

*EM, erythema migrans; LD, Lyme disease. †More than 1 symptom might be reported per case-patient. Rash, any reported, includes EM. Cardiovascular system signs include atrioventricular block, mycarditis, other.

**Figure 3:**
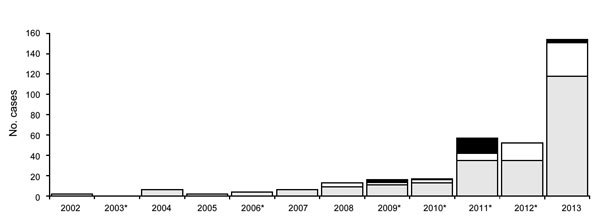
Number of reported Lyme disease (LD) cases, by case classification and year, Nova Scotia, Canada, 2008–2013. Black indicates probable cases—clinical illness and positive serology (2008–2013). White indicates probable cases—clinician-diagnosed erythema migrans and exposure to LD-endemic region (2008–2013). Gray indicates confirmed case—erythema migrans or other clinical illness and positive serology (2002–2007); previous definition plus exposure to LD-endemic region (2008–2013). Asterisk indicates years when LD-endemic regions were declared.

A total of 307 (93%) case-patients reported living in or traveling to an endemic region in Nova Scotia (Figure 1). Of the remaining case-patients, 17 were infected outside the province including patients who traveled to Europe and the United States; for 5 case-patients, the location of exposure was either unknown or outside of regions where LD is known to be endemic. Most (73.3%) cases met the definition for a confirmed case. Most (71.4%) case-patients lived in DHA 1. The number of cases increased substantially during 2012–2013, attributable to increased reports of cases associated with known endemic regions (Figure 3).

A total of 263 (80%) cases had serologic evidence of infection. Of the 66 cases classified as “probable: EM + endemic exposure” that were reported to Nova Scotia public health professionals, 10 did not have any information about laboratory testing, 42 did not have serologic testing, and 14 had serologic test results that were negative by the 2-tier algorithm. Of these 14 patients, 3 were negative on the whole-cell EIA, and 11 were positive by the C6 ELISA but negative by WB.

The annual number of serologic tests for LD in Nova Scotia increased from 1,659 in 2010 to 2,421 in 2013. Testing rates varied by DHA (Figure 1), ranging from 69.1 tests per 100,000 population in DHA 8 to 1,581.9 per 100,000 population in DHA 1 in 2013. Serologic incidence of LD in 2013 varied by DHA, ranging from 0 cases per 100,000 population in DHA 8 to 206.2 per 100,000 population in DHA 1.

### Serosurvey Results

Of 1,855 serum samples tested for antibodies to *B. burgdorferi*, 215 (11.6%) screened positive by the whole-cell EIA (Walpole Laboratories, Walpole, MA, USA) and were sent to the NML for confirmatory testing. Of these, 17 (0.9% of total serosurvey) were positive or equivocal by the C6 ELISA. None were positive by IgG WB using Centers for Disease Control and Prevention (CDC) criteria, but 2 samples were borderline positive (Table 3). If we used CDC criteria for WB interpretation, the estimated seroprevalence of LD in Nova Scotia was 0% (95% CI 0.00%–0.20%). If we considered the 2 borderline WB results or the positive C6 ELISA as evidence of exposure to *B. burgdorferi*, the estimated seroprevalence was 0.14% (95% CI 0.02%–0.51%) and 0.98% (95% CI 0.56%−1.60%), respectively (Table 4).

**Table 4:** LD test results from serosurvey and seroprevalence estimates, Nova Scotia, Canada, 2002–2013*

Region (no. endemic regions)	Laboratory test type					Seroprevalence estimate	
	Total screening tests, whole-cell EIA	Whole-cell EIA positive or indeterminate, no. (%)	C6 positive or equivocal, no. (%)	IgG WB borderline, no. (%)		IgG WB borderline, % (95% CI)	C6 ELISA, %, (95% CI)
DHA 1 (2)	191	21 (11.0)	1 (0.5)	0		0 (0–1.9)	0.52 (0–2.9)
DHA 2 (2)	199	19 (9.5)	2 (1.0)	0		0 (0–1.8)	1.01 (0.1–3.6)
DHA 3	261	42 (16.1)	2 (0.8)	0		0 (0–1.4)	0.77 (0.1–2.7)
DHA 4	120	12 (10.0)	1 (0.8)	1 (0.8)		0.83 (0–4.6)	0.83 (0–4.6)
DHA 5	44	1 (2.3)	1 (2.3)	0		0 (0–8.0)	2.27 (0.1–12.0)
DHA 6 (1)	74	3 (4.1)	0	0		0 (0–4.9)	0 (0–4.9)
DHA 7	72	3 (4.2)	0	0		0 (0–5.0)	0 (0–5.0)
DHA 8	201	22 (10.9)	1 (0.5)	0		0 (0–1.8)	0.5 (0–2.7)
DHA 9 (1)	693	92 (13.3)	9 (1.3)	1 (0.1)		0.14 (0–0.8)	1.3 (0.6–2.5)
Nova Scotia†	1,855	215 (11.6)	17 (0.9)	2 (0.1)		0.14 (0.02–0.51)	0.98 (0.56–1.60)

*DHA, District Health Authority; EIA, enzyme immunoassay; LD, Lyme disease; WB, Western blot. †Provincial seroprevalence estimates weighted by age, sex, and DHA, accounting for oversampling in DHAs 1, 2, and 3.

## Discussion

LD is emerging in Nova Scotia, and public health surveillance data have been useful for characterizing risk and describing the clinical presentation of LD. Most cases are characterized by early localized disease. Compared with data from the United States, Nova Scotia case-patients were less likely to have EM reported as the presenting manifestation (69% vs. 53%) (*11*). Although published studies report that up to 80% of LD case-patients have EM, these findings were in the context of a vaccine trial or active laboratory-based surveillance study in a hyperendemic region where patients were followed closely and physicians were very experienced in diagnosing EM (*12*,*13*). The public health surveillance system in Nova Scotia captures both clinician-diagnosed EM and other skin rashes. Although almost 90% of case-patients reported a rash, only 53% had clinician-diagnosed EM. However, some of the other skin rash cases are also likely to represent EM. Central clearing in only 50% of EM lesions was consistent with other reports that EM might have diffuse erythema or enhanced central erythema rather than central clearing (*14*). An increase in the proportion of cases with clinician-diagnosed EM over time suggests physicians have become more aware of and/or better able to diagnose EM. 

The most common manifestation of late LD in Nova Scotia was arthritis. Although up to 60% of untreated LD results in arthritis, earlier recognition and treatment is expected to greatly reduce its frequency (*7*,*15*–*17*). The increase in the proportion of Nova Scotia case-patients reporting recurrent brief episodes of large joint swelling to 32.5% in 2013 is similar to the proportion of US cases of Lyme arthritis (30%) (*11*). The higher proportion of cases with arthritis in 2013 does not necessarily reflect a large proportion of missed cases during earlier years but might be related to misreporting of arthralgia and reporting bias toward serologically confirmed cases, as well as a general increase in the number of cases and duration of LD in Nova Scotia. Further investigation is needed to understand how many case-patients reporting joint symptoms fit the clinical diagnosis of Lyme arthritis. 

As in the United States, other manifestations, including atrioventricular block and neuroborreliosis, were uncommon (*11*). Only 26% of LD patients in Nova Scotia recalled a tick bite, consistent with US data where 84% and 86% of patients with EM and Lyme arthritis, respectively, failed to recall exposure to blacklegged ticks (*18*–*21*).

The incidence of LD in Canada has increased during the last decade (*2*,*4*), and Nova Scotia has the highest reported incidence (16 cases/100,000 population in 2013). Incidence varies greatly geographically; 1 health region (DHA 1) reported case rates of ≈200/100,000 population in 2013, which is higher than those in northeastern US states (*11*). The increased incidence most likely resulted from the increased number of established blacklegged tick populations; increases in the size, geographic range, and pathogen prevalence within these populations; increasing rates of human infection; and better identification and reporting of LD. Infected blacklegged ticks can be sporadically identified in other regions of the province, but only 1.5% of LD cases were not linked to known endemic regions, suggesting the risk remains localized. Our serosurvey did not identify any persons with *B. burgdorferi* antibodies based on CDC criteria, and even if the 2 borderline results were included, the estimated provincial seroprevalence was much less than 1%. Even in DHAs known to contain endemic foci of LD, we failed to detect any positive serum and found an estimated seroprevalence of <2% in the DHA with the highest reported LD incidence. *B. burgdorferi* seroprevalence in the northeastern United States, with similar environmental conditions to Nova Scotia, ranges from 0% to 18.8% (*22*,*23*). The fact that tick populations endemic to the northeastern United States have been established for much longer than in Nova Scotia might explain this difference. Widespread clinical testing, coupled with the serosurvey results, suggests LD cases most likely are not being missed in Nova Scotia.

Although all of the serum used for the serosurvey was negative by 2-tier testing, 8% of serum samples positive by whole-cell EIA (17/215) were positive on the C6 ELISA. Although some data suggest that classifying LD on the basis of C6 alone is more sensitive than the 2-tier algorithms (*24*), the potential exists for false-positive and false-negative results. The literature suggests that the C6 ELISA specificity is 98.4%–98.6% (*24*,*25*). However, when used as the supplemental test to positive whole-cell lysate EIA, as in our study, its specificity is estimated at 99.1%–99.8% (*26*). The 17 positive C6 results in our study could be in keeping with false-positive results, with this reported specificity. Alternatively, these reactive C6 serum samples could represent patients infected with *B. burgdorferi* and treated early with appropriate antimicrobial drugs. However, because the serum used could not be linked to patients, we have no clinical information to support this possibility. Data suggest that the C6 ELISA has a higher sensitivity for early infection and can be positive before the IgG WB completely matures to include the required 5/10 bands (*27*). In addition, evidence suggests that patients treated early in infection abort the seroconversion response, and a positive IgG WB might not develop (*27*,*28*). Another possibility is that the positive C6 ELISA results from cross-reactivity with another *Borrelia* species, such as *B. miyamotoi*, which has been identified in blacklegged ticks in the United States and Canada, including Nova Scotia (*29*,*30*). Using EIA and WB assays specific for *B. miyamotoi*, Krause et al. found serologic evidence of acute infection in patients living in endemic regions who had a viral-like illness (*31*). Although a recent study found 2 of 34 ticks from Nova Scotia submitted as part of passive surveillance had positive PCR for *B. miyamotoi* (*30*), no data are available that examined the potential cross-reactivity of whole-cell sonicate or C6 *B. burgdorferi* EIAs with patient serum containing antibodies to *B miyamotoi*. If these hypotheses are correct, we should have seen a disproportionate number of positive serum samples from regions with the highest risk for exposure to infected blacklegged ticks, such as DHA 1, where an infected population of blacklegged ticks has been established since 2003 and where most case-patients reside and were exposed. However, seroprevalence of C6 positive serum did not differ significantly among any of the DHAs.

Our study has several limitations. Our clinical data are limited to the case report forms used by Nova Scotia public health professionals; thus, the precision regarding the clinical presentation is limited. For example, the inclusion of categories of influenza-like illness is not specific and could represent a respiratory illness. Furthermore, the case reports do not always differentiate between single EM and multiple EM, so classification of early localized and early disseminated infection was not possible. Early LD, the most common presentation, is predominantly a clinical diagnosis for which sensitivity of serologic testing is poor (<50%) and not recommended. Thus, surveillance data might underestimate incidence if not all clinical cases are captured. We recognize that estimating the extent of underreporting is difficult and that underreporting could vary geographically. However, in the cohort reported here, 42 of 66 cases classified as “probable: EM + endemic exposure” followed the Infectious Diseases Society of America guidelines (*7*) and were not tested serologically. These reports have come from 5 different districts across the province suggesting that physicians are reporting at least some *B. burgdorferi* infections on the basis of clinical presentation alone.

Another limitation is that the serosurvey samples might not be representative of the population at risk. We used samples of residual serum from diagnostic testing that might be biased toward a population with more medical co-morbidities or different risk and health-seeking behaviors (*32*). Our study attempted to reduce this bias by selecting samples that were originally collected for routine diagnostic testing, with the aim of capturing persons undergoing routine well-person screening. However, this sample will exclude persons who generally do not access regular medical care. Despite this limitation, the sampling method has been used for other infections and is thought to provide an acceptable balance between representativeness and feasibility (both practical and financial). In fact, the only published study that has compared the residual serum approach with population-based sampling yielded comparable estimates of immunity against 5 vaccine-preventable diseases, with an ≈7-fold increased cost for the population-based approach (*33*). Still, risk for exposure to blacklegged ticks through outdoor activity in endemic regions was unavailable for serosurvey specimens and probably has greater heterogeneity than probability of vaccination. Also, our sample did not include the 0–9- or >65-year age groups.

Although LD incidence is increasing in Nova Scotia, infections appear to be restricted to regions within the province where populations of infected blacklegged ticks are known to be endemic. These findings support a targeted approach to public health risk messaging. Our seroprevalence study suggests that <1% of Nova Scotia residents have been exposed to *B. burgdorferi*. However, as tick populations continue to expand, we expect LD rates to continue to increase. Residents of, and travelers, to Nova Scotia need to be vigilant and take precautions to reduce their risk for LD when they venture into regions where ticks are present. Because only a minority of patients will report a tick bite, physicians should be aware of the manifestations of LD and consider it when patients have compatible symptoms and exposure to an endemic region, through residence or travel, in Nova Scotia.
